# Comparative assessment of root exudation in maize: Influence of experimental setup, growth conditions and root hairs

**DOI:** 10.1007/s11104-026-08324-x

**Published:** 2026-02-05

**Authors:** Michael Santangeli, Anna Heindl, Lisa Stein, Alice Tognacchini, Eva Oburger

**Affiliations:** Department of Ecosystem Management, Climate and Biodiversity, Institute of Soil Research, BOKU University, Tulln an Der Donau, 3430 Austria

**Keywords:** Root exudates, Dissolved Organic Carbon, Field, Hydroponic, Soil-based experiments

## Abstract

**Background and aims:**

A major challenge in root exudation research is obtaining exudates samples that accurately reflect the exudation processes under natural soil growth conditions. Both growth environment and experimental setup can significantly influence root exudation dynamics. This study investigated how different experimental systems and growth conditions affect carbon exudation in maize (*Zea mays* L.) roots and whether these factors could influence the detection of genotypic differences between the wild type (B73) and its hairless mutant, *rth3.*

**Methods:**

Maize plants were grown under various experimental conditions, including soil-based and hydroponic systems. Root exudates were collected using a combination of traditional and innovative sampling approaches. Carbon exudation rates were compared across experimental setups and genotypes. Laboratory results were further compared with data from a separate field experiment.

**Results:**

Exudation rates obtained from soil-based laboratory experiments were comparable to those observed in the field under similar growth temperatures. The contribution of root hairs to total carbon exudation was negligible compared to the effect of growth conditions and experimental setup. Large differences in root biomass introduced bias into exudation measurements, particularly when root to sampling volume ratio (RSVR) varied substantially.

**Conclusions:**

Experimental setup and environmental conditions have a strong influence on root exudation measurement. Soil-based laboratory systems that closely replicate field conditions, particularly temperature, can serve as reliable proxies for field experiments, providing ecologically meaningful data. Maintaining a consistent RSVR is also essential for obtaining accurate and comparable results. These findings offer important methodological guidance for reliably quantifying root carbon exudation in maize

**Supplementary Information:**

The online version contains supplementary material available at 10.1007/s11104-026-08324-x.

## Introduction

Root exudates play a central role in shaping rhizosphere processes, influencing nutrient cycling, microbial interactions, and plant performance (Bais et al. 2006). Root exudates constitute a significant and highly dynamic source of dissolved organic carbon (DOC) in the rhizosphere, alongside other inputs such as microbial products and soil organic matter decomposition (Neff and Asner 2001). Obtaining ecologically meaningful root exudation data is a crucial step towards a better understanding of the regulatory role of root exudates in the rhizosphere processes. However, due to the complexity of soil structure, root architecture, and the processes immediately triggered when plant roots release carbon compounds into the soil (such as sorption, changes in solution speciation, and microbial decomposition), sampling exudates from soil-grown plants is extremely challenging (Oburger and Jones 2018; Otxandorena-Ieregi et al. 2024; van Dam and Bouwmeester 2016).

The specific advantages and disadvantages of the methods commonly used to sample root exudates have been extensively discussed in detail elsewhere (e.g., Escolà Casas and Matamoros 2021; McLaughlin et al. 2025; Oburger and Jones 2018; Vranova et al. 2013; Williams et al. 2021). Due to its simplicity compared to soil-based techniques, the hydroponic-based setup has been widely used in rhizosphere research to sample root exudates (Aleksza et al. 2024; Kawasaki et al. 2018; Neumann and Römheld 1999; Otxandorena-Ieregi et al. 2024; Zhalnina et al. 2018). In hydroponics, a nutrient solution is used as the growth medium without a solid substrate, allowing direct access to the roots for the collection of exudates, without the need of separating the roots from a growth substrate. This avoids root damage, minimises the influence of microorganisms and disturbance of the soil matrix. However, the lack of the physical and biological complexity intrinsic to the soil environment can alter plant physiology and root architecture (Ascough and Fennell 2004; Neumann et al. 2014; Ostonen et al. 2007), raising concerns about the comparability of exudates collected in hydroponic systems with those from soil-grown plants (Oburger and Jones 2018).

In contrast, soil-based systems more closely mimic plants natural growth environment, enabling a more ecologically relevant assessment of root exudation dynamics. However, the inherent heterogeneity of the soil environment makes it more challenging to standardise experimental procedures and to control plant growth. Factors such as soil texture, mineral composition, variability in water and nutrient availability, and variation in microbial community structure can shape the exudation profile (Jacoby et al. 2017; Oburger et al. 2013; Sasse et al. 2020; Zhao et al. 2021), as well as influence root morphology and architecture (Bengough et al. 2006; Neumann et al. 2014; Ostonen et al. 2007). Moreover, processes such as sorption and microbial degradation can further complicate the quantification and interpretation of root exudation dynamics in soil (Phillips et al. 2008; Williams et al. 2021). These overlapping effects make it difficult to disentangle specific causal factors, potentially masking the effect of a specific treatment or experimental variables. This is particularly relevant when investigating the contribution of root hairs to root exudation. Research shows that root hairs increase the root surface area available to absorb nutrients and release exudates (Holz et al. 2017), which in turn favours root-soil contact (Cai and Ahmed 2022). However, their contribution in nutrient uptake may be less significant in environments where nutrients are readily available in the growth medium, such as hydroponics.

Nonetheless, determining the contribution of root hairs to the total root exudation is a complex endeavour. While the hydroponic system has its own limitation, the utilisation of a soil-hydroponic hybrid approach, which involves a root washing step of soil-grown roots prior to hydroponic exudate collection, can potentially cause physical damage or rupture of the root hairs during the washing procedure (Oburger and Jones 2018). This disturbance can potentially alter exudation patterns and affect the gross carbon exudation rate. Hence, sophisticated designs and minimally invasive sampling techniques are needed to reduce disturbance while maintaining ecological relevance. In this regard, the rhizobox system designed by Wenzel et al. (2001), and further implemented with the automatic exudate collector system by Oburger et al. (2013), can represent an effective solution. This system keeps the root and soil in separate compartments yet facilitates nutrient and water exchange through a nylon membrane and can be considered as a valuable reference, allowing the sampling of root exudates from soil-grown plants with minimal disturbance.

A central question in root exudation research, alongside the comparability of exudate results from soil versus hydroponically grown plants, is whether results obtained under controlled laboratory conditions can reliably represent the exudation patterns observed in field environments (Heuermann et al. 2023; Oburger and Jones 2018). Heuermann et al. (2023), in a recent study on cover crops, has shown that while some exudates are common across soil and hydroponic systems, others are system-specific. These findings highlight the importance of conducting comparative studies to evaluate the ecological relevance and transferability of exudation data generated under artificial conditions.

The primary aim of this study is to assess how different experimental setups and growth conditions affect carbon exudation in maize (*Zea mays* L.) plants. To this end, we compared three complementary experimental setups: (i) a hydroponic system, (ii) a soil column experiment (SCE) combined with the soil-hydroponic sampling approach, and (iii) a rhizobox system integrated with an automated exudate collector (soil-REC). To place these results in a broader context, we further compared them with field data obtained using the same maize genotypes and substrate, as reported in Santangeli et al. (2024). We hypothesized that the rate of carbon exudation is strongly influenced by the experimental setup, and that the comparability of exudation rates between field and soil-based laboratory experiments will depend on how closely the environmental growth conditions align.

This study focuses on total carbon exudation rather than individual metabolites or metabolite classes. Gross carbon exudation is a critical, yet frequently overlooked, parameter that ultimately determines the amount of carbon allocated to the rhizosphere. While specific metabolite profiles can vary significantly depending on environmental and experimental factors, total carbon exudation provides a comprehensive view of carbon flux to the soil. This makes it possible to identify and interpret broader patterns that might otherwise remain undetected if only individual compounds are considered.

Finally, we aimed to cross-validate previous findings on the contribution of root hairs to carbon exudation (Lohse et al. 2023; Santangeli et al. 2024), where the *Zea mays* L. root hairless mutant *rth3* showed a higher exudation rate compared to its wild-type sibling. The soil-REC system, specifically developed to collect root exudates from soil-grown plants without damaging the root system (or root hairs) (Oburger et al. 2013), was used to minimize artefacts caused by root damage. This design allowed us to test the hypothesis that potential root hair damage in the soil-hydroponic hybrid approach has a minor effect on carbon exudation rate compared to the influence of growth conditions and experimental setup.

## Material and methods

**Fig. 1:**
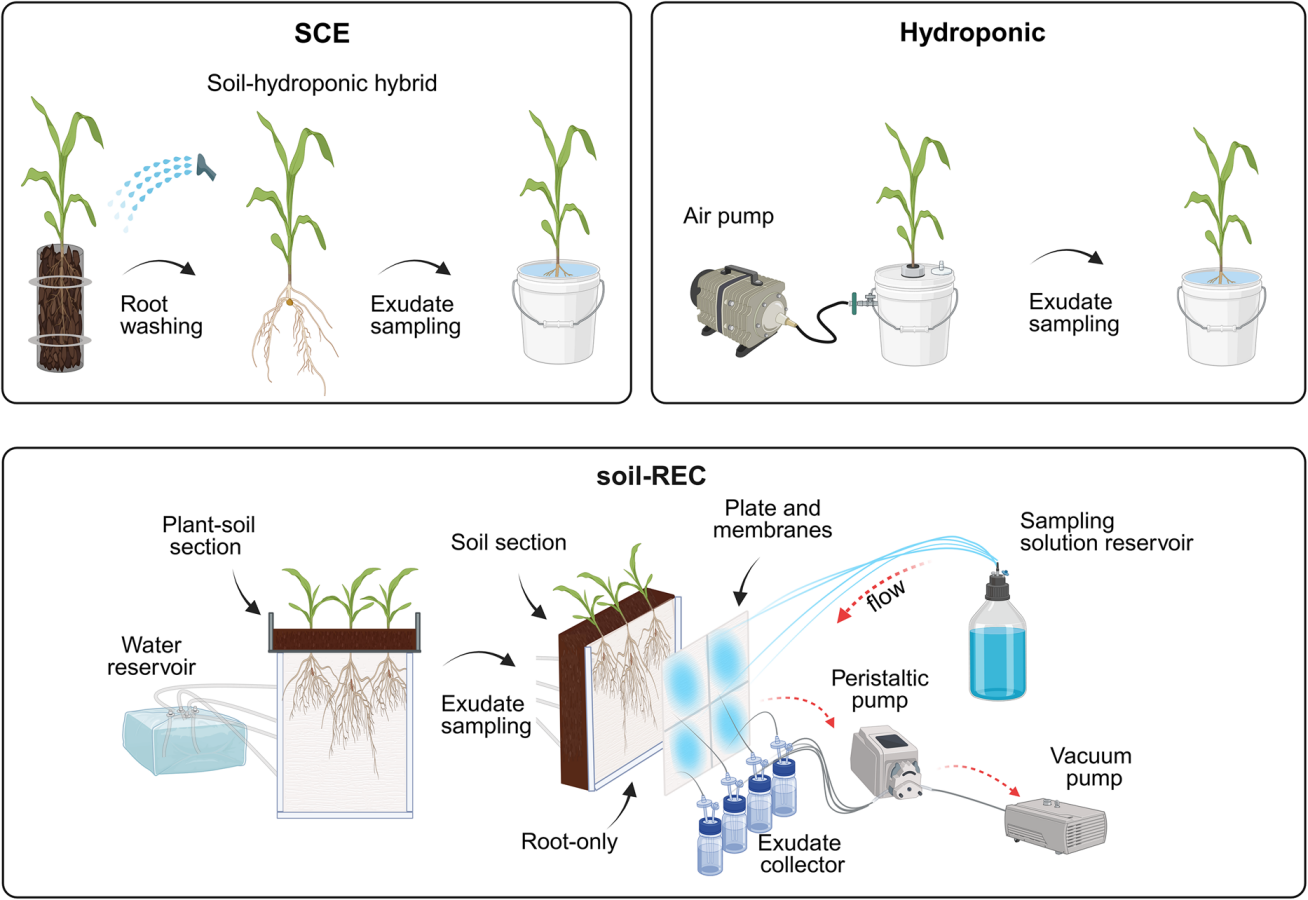
Schematic overview of the laboratory experimental systems and the different root exudate sampling approaches used in this research. SCE (soil column experiment; soil-hydroponic hybrid exudate sampling): plants were grown in soil and, after careful root washing, transferred to a hydroponic system for exudate sampling. Hydroponic system: plants were grown and sampled entirely under hydroponic conditions. Soil-REC (soil root exudate collector): plants were grown in the plant-soil section, with the root system extending into a soil section where roots grew on a membrane in two-dimensions (2D); root exudate sampling was performed using an automated root exudate collector operating under vacuum through a system of peristaltic pumps in cyclic sampling mode. Created in BioRender. Santangeli, M. (2026) https://BioRender.com/8lbjt9x

In this study, three distinct root exudate sampling methods (i.e., soil-hydroponic hybrid, hydroponic, and exudate collector) and four experimental setups (i.e., soil column, hydroponic, soil-REC, and field column) were utilized to cultivate maize plants and collect root exudates (Fig. 1). The laboratory experiments were conducted in controlled growth cabinets equipped with LED lighting (Percival Scientific), while the field column experiment was carried out under natural field conditions.

### Plant material and growth conditions

The study employed two genotypes of maize (*Zea mays* L.), namely wild-type (WT) and the *rth3* root hairless mutant (Wen and Schnable 1994). Although the shoot phenotype of the *rth3* mutant does not appear to be affected by the lack of functional root hairs, the yield was 20–40% lower than the wild type under field conditions (Hochholdinger et al. 2008; Vetterlein et al. 2021). Maize seeds were surface sterilised with 10% H_2_O_2_ for 10 min, rinsed 4 times with deionised water (DI), and then soaked in saturated CaSO_4_ for 3 h before sowing (Lippold et al. 2021). The plants were grown in growth cabinets with a photoperiod of 12 h, a daytime temperature of 21 °C and 18 °C at night. Humidity was maintained at 65%, and the light intensity was set to 350 µmol m^−2^ s^−1^ of Photosynthetic Active Radiation (PAR) according to Vetterlein et al. (2021). The soil column experiment (SCE) and the hydroponic experiments were repeated with a daytime temperature of 28 °C and 21 °C at night to better mimic the temperatures experienced by the plants grown in the field (Jorda et al. 2022). All the laboratory experiments included six replicates of both maize genotypes, which were grown for 21 days and harvested on day 22 after sowing, aiming to reach the growth stage BBCH 14 (four unfolded leaves), as defined by the BBCH scale (Bleiholder et al. 2001).

### Soil substrate

The substrate used for both the SCE and the rhizobox-collector experiment (soil-REC) was a haplic Phaeozem soil derived from the top 50 cm of an agricultural field in Schladebach, Germany (51°18′31.41′′ N; 12°6′16.31′′ E). Further information on the loam substrate can be found in Vetterlein et al. (2021). Prior to fertilisation, the soil was progressively sieved, starting with a mesh size of 4 mm, then 2 mm and finally 1 mm. The substrate was fertilized according to Lippold et al. (2021) to achieve a target nutrient concentration of 50 mg kg^−1^ for nitrogen (N) and potassium (K), 25 mg kg^−1^ for magnesium (Mg) and 40 mg kg^−1^ for phosphorus (P), supplied as NH_4_NO_3_, K_2_SO_4_, MgCl_2_ × 6 H_2_O and CaHPO_4_ respectively. The fertilisation regime was established to maintain phosphorus concentrations in the WT genotype below the threshold of adequate supply (Lippold et al. 2021). The substrate of each soil column and rhizobox was fertilised individually. N, K and Mg were applied as solutions, after which the soil was mixed manually and left to air dry for 72 h. P was added as powder after the drying phase. Soil was further sieved to a particle size of 1 mm before filling the columns and rhizoboxes to remove aggregates formed during fertilization.

### Soil column experiments (SCE)

The SCE was carried out in accordance with Lippold et al. (2021), with some modifications. Briefly, the plants were grown in cylindrical acrylic glass tubes (25 cm height × 7 cm diameter), filled with the loam substrate up to 23 cm, with a bulk density of 1.26 g cm^−3^. The tubes were cut into two halves and held together by two metal fastening rings to facilitate access to the root system during the washing procedure. The bottom of the column was closed with a 30 µm nylon mesh (SEFAR Nitex, Heiden, Switzerland), fixed with tape and an acrylic ring. A representative sketch of the soil column design can be found in Fig. 1. The seeds were planted at a depth of 1 cm, one seed per column. A 50 g gravel layer was placed on top of the soil surface to prevent damage from irrigation and reduce evaporation. The columns were placed in irrigation trays and watered to achieve a volumetric water content of 22%. The initial irrigation was conducted by applying one-third of the water (DI) from the top, while the remaining two-thirds were supplied from the bottom. Subsequently, each column was weighed to determine the total weight. Throughout the growth period, the columns were rewatered to their initial weight, with two-thirds of the water being added from the top and one-third from the bottom. Plants were watered at 2, 5, 9, 13, 15, 17, 19, and 21 days after sowing.

### Hydroponic experiment

Maize plants were cultivated in a hydroponic system using polypropylene buckets (16 cm height × 13 cm diameter) with lids. Each bucket was equipped with a connector for an air pump to facilitate the continuous aeration of the nutrient solution. The buckets were filled with 1.8 L of nutrient solution. The lids were modified to include two holes: a smaller one for an outlet filter and a larger one to accommodate a cut polystyrene foam disc, which served as a seedling holder. After sterilization (Section "Plant material and growth conditions") the seeds were placed on moist tissue paper in closed Petri dishes and kept in the dark until germination. Five days after germination, seedlings were transferred to the hydroponic system (one per bucket), using a modified half-strength Hoagland nutrient solution (Hoagland and Arnon 1950) consisting of 3 mM KNO_3_, 0.5 mM NH_4_H_2_PO_4_, 2 mM Ca(NO_3_)_2_, 1 mM MgSO_4_, 7.5 µM MnCl_2_, 0.25 µM Na_2_MoO_4_, 25 µM H_3_BO_3_, 0.15 µM CuSO_4_, 0.37 µM ZnSO_4_, 62.5 µM Fe-EDTA. The nutrient solution was changed three times during the growth period, on days 12, 16, and 20.

### Rhizobox-collector experiment (soil-REC)

The rhizobox design used for this experiment was previously described in Wenzel et al. (2001) and adapted by Oburger et al. (2013) to include a root exudate collector system. A schematic overview of the system is presented in Fig. 1. Briefly, the rhizobox was composed of three separate compartments assembled: i) plant-soil, ii) soil and iii) root-only. The plant-soil compartment was placed on top of the soil compartment and had a 2 mm wide slot at its base, which allowed the root to grow downwards along the rhizo compartment, separated from the roots by a 30 µm nylon mesh (SEFAR Nitex, Heiden, Switzerland). While physically separating roots from the soil, the nylon mesh still allowed the exchange of solutes between root and soil. Further details about the experimental design, including diagrams and construction information, can be found in Wenzel et al. (2001). The fertilized and sieved loam soil (see Section "Soil substrate") was used to fill the soil compartment to reach a bulk density of 1.26 g cm^−3^ and watered to 22% volumetric water content. Six maize seeds were sterilised (as described in Section "Plant material and growth conditions") and placed in the plant-soil compartment, then covered with a layer of gravel. Soil moisture was maintained at a constant level by inserting 4 nylon cords (enclosed in polyethylene tubes to prevent evaporation) into the soil on one side and into a DI water tank on the other, ensuring continuous water supply (Fig. 1).

### Field plot experiment

The field experiment was conducted at the Bad Lauchstädt (Germany) research station (51°22′0′′ N, 11°49′60′′ E) (Vetterlein et al. 2021). Details of the experimental design are described in full by Santangeli et al. (2024). Briefly, custom-designed PE tubes, referred to as soil columns (30 cm height × 20 cm diameter, with 5 cm-wide drilled holes), were assembled and placed in the field plot. The inner walls of each soil column were lined with a 30 µm nylon mesh (SEFAR Nitex, Heiden, Switzerland) to allow the exchange of water, solutes, and microorganisms while preventing root growth beyond the mesh barrier. The columns were filled with the same substrate used in the SCE and soil-REC experiments. In April 2019, plants were sown under field conditions and sampled during the 2019 growth season at BBCH growth stages 14 (four unfolded leaves) and 19 (nine or more unfolded leaves) (Santangeli et al. 2024). A single maize seed was planted in the soil column for BBCH 19, while two maize seeds were planted in the BBCH 14 columns to ensure sufficient root biomass at the earlier growth stage.

### Root exudate sampling

An overview of the exudate sampling procedure for the respective experiments is provided in Fig. 1. Briefly, in SCE and in the field experiment, exudates were sampled by using a soil-hydroponic hybrid approach (Oburger et al. 2014; Oburger and Jones 2018). Roots were carefully washed in tap water to remove soil particles and then soaked three times in DI water, each for approximately 5 min, for osmotic adjustment and to minimize potential cell leakage resulting from root damage during the sampling procedure. Following this, roots were soaked for 1 h in 0.5 L DI water as a recovery period and then finally transferred to the final sampling solution consisting of 0.01 g L^−1^ Micropur classic (Katadyn®, Switzerland) dissolved in Milli-Q water for 2 h. The Micropur was added as a microbial growth inhibitor to prevent metabolite degradation by microbes during the sampling period (Otxandorena-Ieregi et al. 2024). The hydroponic experiment was sampled using the same procedure, except for the root-washing step. Exudate samples were then vacuum filtered with a 0.2 µm cellulose acetate membrane (OE 66, Whatman, UK) using vacuum solvent filter degasser systems connected to a vacuum pump, split up into aliquots and frozen at −20 °C until analysis.

The design and functionality of the root exudate collector employed in the rhizobox experiment are described in detail in Oburger et al. (2013). Briefly, the exudates were collected using an acrylic plate covered by a 0.2 µm cellulose acetate membrane (OE 66, Whatman, UK) placed directly on the root mat growing along the nylon membrane in the root section of the rhizobox. The plate featured four vacuum-tight sections to facilitate membrane rinsing and evenly distribute sampling solution across the membrane surface. The plate was equipped with a double membrane layer: an inner layer with a 100 µm mesh to ensure uniform water flow and distribution along the plate, and an outer layer consisting of a 0.2 µm cellulose acetate membrane (OE 66, Whatman, UK). During sampling, the plate was gently pressed against the root mat using spring screws fixed to the rhizobox frame. Exudate collection was achieved by connecting each section of the plate to a sampling solution reservoir and to four different glass sampling vials covered with aluminium foil, one for each section of the plate. Each plate section had one inlet from the solution reservoir and one outlet to the sampling vials. The system operated under constant vacuum, with a peristaltic pump rinsing the membrane every 4 min. Each rinsing cycle lasted 3 min, and the four sections were rinsed sequentially.

Prior to the exudate collection, the plates were flushed with three cycles of the sampling solution, which contained 0.01 g L^−1^ Micropur (Katadyn®, Switzerland). The root mat was then rinsed for 1 h with DI water, followed by three additional cycles with the sampling solution to flush the inlet and outlet tubing. Root exudates were subsequently collected over a period of approximately 2.5 h at the same daytime (i.e., starting at 11:00 AM) as the other experiments to avoid any variation related to diurnal changes. The four sampling vials from each replicate were pooled together, divided into aliquots, and stored at −20 °C until analysis.

### Root morphology and plant biomass

After root exudate sampling, an aliquot of fresh root material was taken from each sample and stored in 50% (*v/v*) water–ethanol solution at 4 °C for WINRhizo analysis. The remaining root aliquot and the shoot biomass was oven-dried at 60 °C for 72 h. The root aliquots stored in ethanol were then scanned at 600 dpi using a flatbed scanner (EPSON Perfection V700). The resulting images were analysed with WinRHIZO Pro™ (Version 2007 d, Regent Instruments, Canada). Root traits (i.e., total root length, total surface area and root average diameter) were obtained by extrapolating the values of the WinRHIZO aliquot to the whole root system (calculated as dry mass of WinRHIZO aliquot + remaining roots biomass) (Santangeli et al. 2024). The root to sampling volume ratio (RSVR) was calculated as the dry root biomass divided by the exudate sampling volume (g L^−1^) (Otxandorena-Ieregi et al. 2024).

### Quantification of dissolved organic carbon

Dissolved organic carbon (DOC) was measured photometrically following the protocol of Oburger et al. (2022). Briefly, a calibration curve was generated using potassium phthalate (KHP, Elementar 35.00–0151) standards ranging from 0.5 to 40 mg C L^−1^ prepared in milli-Q water (MQW). Calibration blanks, standards, exudate samples, and sample blanks (i.e., the exudate sampling solution only) were pipetted into 96-well microplates (Greiner UV-STAR® flat-bottom) suitable for absorbance measurements within the UV/VIS spectrum. Absorbance was recorded at 260 nm using a TECAN Infinite® 200 PRO nano plate reader (Tecan, Switzerland). The DOC concentrations in the soil-REC samples were below the limit of quantification. Therefore, 1.4 mL of each sample was freeze-dried (Alpha 1–4 LSCplus, Christ, Osterode am Harz, Germany) and then resuspended in 0.5 mL of MQW for DOC measurement. Carbon exudation rates of soil column and hydroponic experiments were calculated based on root surface area (RSA) per hour, while soil-REC exudation rate was normalised to root projected area (i.e., roots in contact with the membrane).

### Statistical and data analysis

The experimental setup, sampling procedures, and analysis followed a completely randomised design. The laboratory experiments were conducted in six replicates per treatment and included six experimental blanks (four for the soil-REC experiment), while the field experiment was performed in four replicates and four experimental blanks. Values are presented as means ± SEM. When not normally distributed, data were log-transformed prior to statistical analysis. Differences between experimental systems and genotypes were evaluated using the two-way Analysis of Variance (ANOVA). If no significant interaction was found, differences between genotypes were further tested using a T-test. Differences between root to sampling volume ratio (RSVR) were evaluated using ANOVA, while differences between growth temperatures for the various experimental systems and genotypes were assessed using three-way ANOVA. The significance level α was set to 0.05. All statistical tests and graphs were performed using GraphPad Prism 10.3.1 for Windows (GraphPad Software, San Diego, CA, USA). For the BBCH 14 and soil-REC, more than one plant was grown per column and rhizobox, respectively. For these experiments the biomass values are presented as plant average. One *rth3* replicate from the hydroponic experiments at both 21 °C and 28 °C, as well as from the SCE at 28 °C, and one WT replicate from the soil-REC setup, died during the experiment.

## Results

### Effect of experimental system on carbon exudation rate and plant biomass

To assess whether the experimental system influenced the root C exudation in maize, we compared the WT and the hairless mutant *rth3* cultivated under identical growth conditions (21 °C daytime and 18 °C at night). Exudates were collected using different exudate sampling methods tailored to each experimental system, including hydroponic, soil-hydroponic hybrid, and exudate collector approaches (Fig. 1).

**Fig. 2:**
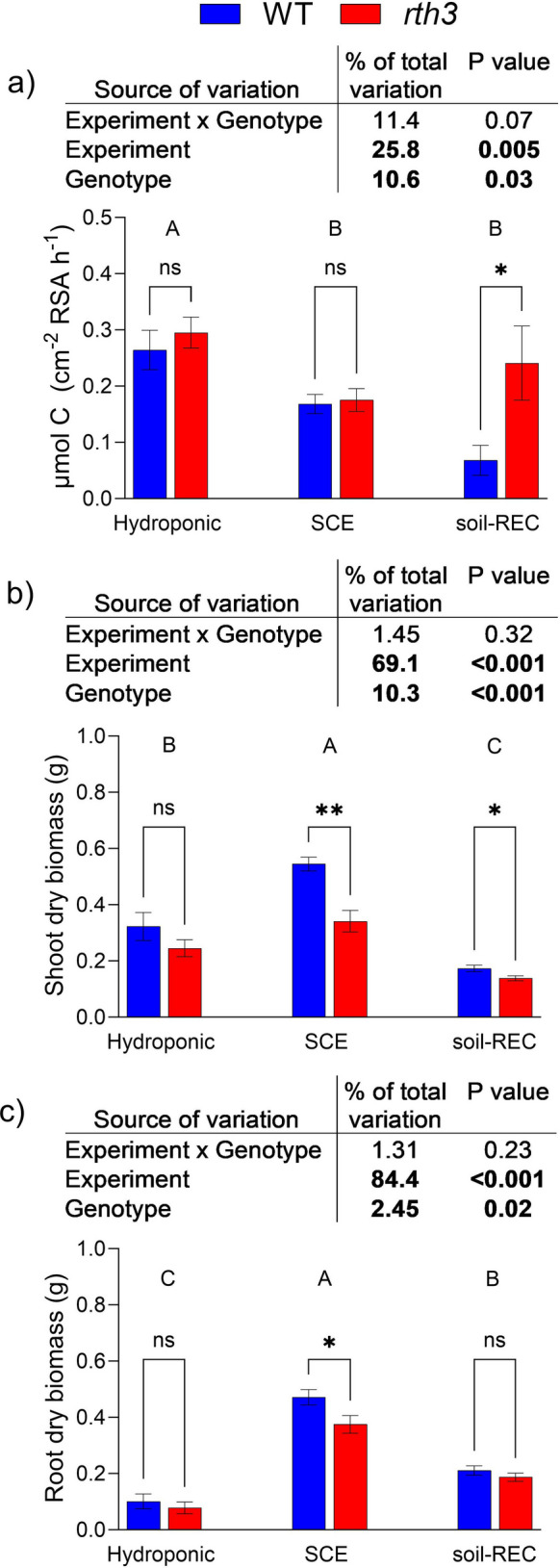
Comparison of hydroponic, soil column (SCE), and rhizobox with exudate collector (soil-REC) systems for wild-type (WT) and root hairless (*rth3*) mutant maize plants grown under the same growth conditions (21 °C (day)/18 °C (night)): **a**) Carbon exudation rate; **b**) Shoot biomass; **c**) Root biomass. Differences between experimental setups were evaluated with two-way ANOVA followed by Tukey’s post hoc test. Differences between genotypes were evaluated by Welch t-test. *** *p* < 0.001, **p < 0.01, * *p* < 0.05, ns = not significant. Different capital letters indicate difference among experimental setups. Values represent means ± SEM

The experimental system had a significant effect on C exudation rate (p = 0.005), accounting for approximately 26% of the total variation in the dataset (Fig. 2a). Overall, C exudation rates in the hydroponic system were significantly higher compared to the two soil-based experimental system (SCE, soil-REC), while no statistically significant difference was observed between SCE and soil-REC (Fig. 2a).

Although no significant differences in C exudation rates between WT and *rth3* were observed in either the hydroponic or SCE, a significant genotypic difference emerged in the soil-REC system (Fig. 2a). A genotype × system interaction accounted for 11.4% of the total variation in exudation, though it was only marginally significant (p = 0.07; Fig. 2c).

**Fig. 3:**
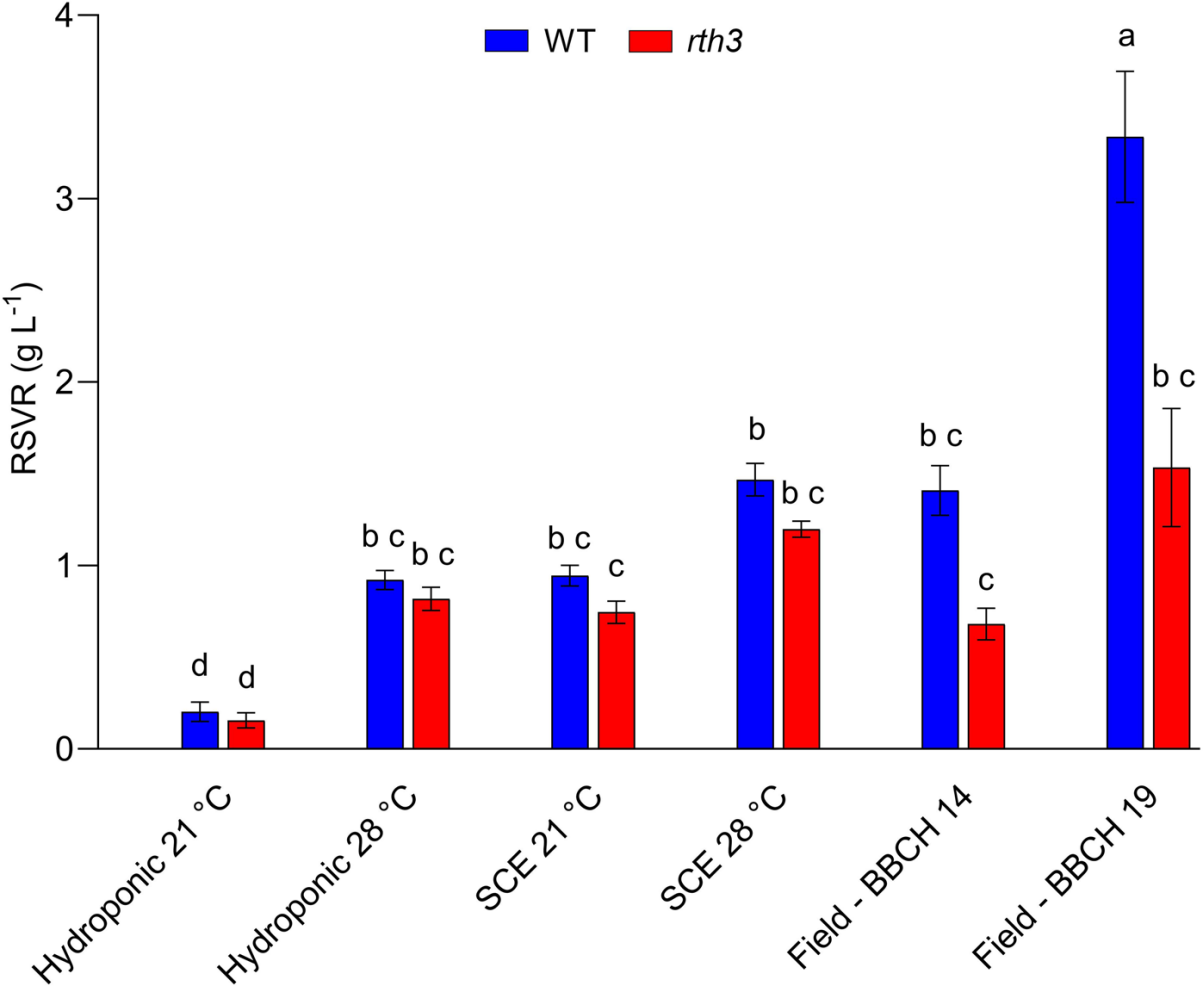
Root to sampling volume ratio (RSVR) across all experimental systems and conditions. Differences were evaluated using ANOVA followed by Tukey’s post-hoc test. Different letters indicate significant differences (*p* < 0.05) between treatments. Values represent means ± SEM

Shoot and root biomass also varied significantly across experimental systems (Fig. 2b, c), likely reflecting to system-specific conditions. Plants grown hydroponically (21 °C) showed reduced shoot and root biomass relative to those in the SCE (21 °C), which also resulted in a significantly lower root to sampling volume ratio (RSVR) (Fig. 3). Due to the specific design of the soil-REC system, it was not possible to accurately estimate RSVR for this setup. In soil-based systems, *rth3* exhibited consistently lower biomass compared to WT, whereas no significant genotypic differences were detected in hydroponically grown plants for either shoot or root biomass (Fig. 2b, c).

### Effect of environmental conditions on carbon exudation rate and plant biomass

To better align controlled laboratory conditions with those observed in the field, and to assess the impact of growth temperature on C exudation, both hydroponic and SCE were repeated with growth temperatures set at 28 °C during the day and 21 °C at night.

**Fig. 4:**
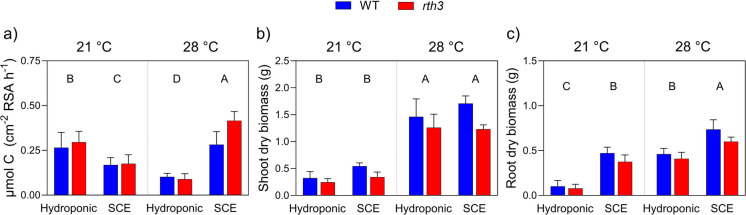
Comparison of hydroponic and soil column (SCE) systems for wild-type (WT) and root hairless (*rth3*) mutant maize under varying temperatures: (**a**) Carbon exudation rate, (**b**) Shoot biomass, and (**c**) Root biomass, were measured for plants grown under two temperature regimes: 21 °C (day)/18 °C (night) and 28 °C (day)/21 °C (night). Differences between experimental setups (i.e., experiment × temperature) were evaluated with two-way ANOVA followed by Tukey’s post hoc test. Different capital letters indicate differences among experimental setups. Values represent means ± SEM

C exudation rates were primarily influenced by the interaction between temperature and experimental system (64% total variation, *p* < 0.001; Table 1). This interaction was driven by the contrasting trends: in hydroponic plants, exudation rates decreased at 28 °C relative to 21 °C, whereas in soil-grown plants (SCE), exudation rates increased under the higher temperature regime (Fig. 4a).

**Table 1:** Results of three-way ANOVA testing the effects of temperature, experimental setup (hydroponic vs. soil solumn, SCE), and genotype (WT vs. *rth3*) on carbon exudation rate, shoot biomass, and root biomass of maize plants

C exudation rate	% of total variation	*P* value	SS (Type III)	DF	F value
Temperature	0.0281	0.82	0.000160	1	0.051
Experiment	10.4	< 0.001	0.0591	1	18.8
Genotype	3.12	0.02	0.0177	1	5.66
Temperature × Experiment	64.1	< 0.001	0.365	1	116
Temperature × Genotype	0.861	0.22	0.00490	1	1.56
Experiment × Genotype	1.86	0.07	0.0106	1	3.38
Temperature × Experiment × Genotype	3.59	0.02	0.0204	1	6.51
Residual			0.116	37	
Shoot biomass	% of total variation	*P* value	SS (Type III)	DF	F value
Temperature	83.1	< 0.001	12.3	1	421
Experiment	1.35	0.01	0.200	1	6.82
Genotype	4.32	< 0.001	0.641	1	21.9
Temperature × Experiment	0.0491	0.62	0.00729	1	0.25
Temperature × Genotype	0.740	0.06	0.110	1	3.75
Experiment × Genotype	0.763	0.06	0.113	1	3.86
Temperature × Experiment × Genotype	0.108	0.46	0.0160	1	0.547
Residual			1.08	37	
Root biomass	% of total variation	*P* value	SS (Type III)	DF	F value
Temperature	41.2	< 0.001	0.970	1	189
Experiment	44.7	< 0.001	0.894	1	174
Genotype	3.02	< 0.001	0.0655	1	12.8
Temperature × Experiment	1.32	0.02	0.0286	1	5.58
Temperature × Genotype	0.791	0.08	0.00307	1	0.60
Experiment × Genotype	0.141	0.44	0.0172	1	3.35
Temperature × Experiment × Genotype	0.00342	0.90	7.43e-005	1	0.015
Residual			0.00513	37

In contrast, growth temperature had a strong influence on plant growth (83% total variation, *p* < 0.001; Table 1). Both shoot and root biomass were significantly higher at 28 °C compared to 21 °C within each respective experimental system (i.e., hydroponic or SCE; Fig. 4b, c). Plants grown in soil (SCE) generally produced more biomass than those cultivated hydroponically. However, no significant difference in shoot biomass was observed between SCE and hydroponic plants at 28 °C (Fig. 4b). Across treatments, hydroponic plants exhibited lower root-to-shoot ratios and smaller average root diameters than soil-based systems, with the exception of SCE at 28 °C, where average root diameter was similar to that observed under hydroponic conditions and specific root length and specific root surface area were higher compared to SCE at 21 °C (Fig. S2).

Genotypic differences were evident in both shoot and root biomass, although they accounted for a relatively small proportion of the total variance (4.3% for shoot and 3% for root biomass, Table 1), with *rth3* plants producing less biomass than WT (Fig. 4b, c). No significant genotype × temperature interaction was found for either trait. This difference was also reflected in higher root surface area and root length in WT plants compared to *rth3*, particularly in soil-based systems (Fig. S2).

**Fig. 5:**
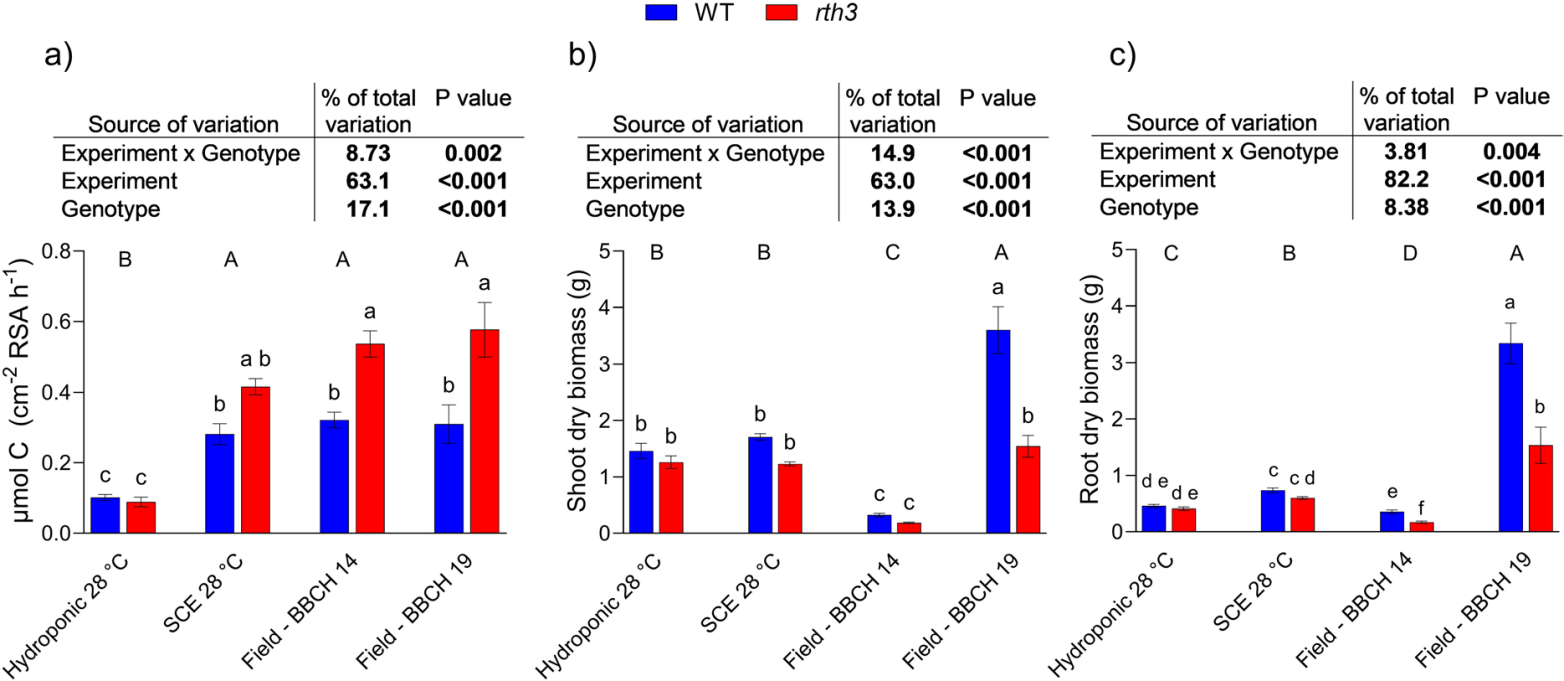
Comparison of hydroponic, soil column (SCE), and field data from Santangeli et al. (2024) for wild-type (WT) and root hairless (*rth3*) mutant maize plants grown under comparable growth conditions: a) Carbon exudation rate; b) Shoot biomass; c) Root biomass. Differences between genotypes (i.e., WT and *rth3*) and experimental setups were evaluated with two-way ANOVA followed by Tukey’s post hoc test. *** *p* < 0.001, **p < 0.01 *, *p* < 0.05, ns = not significant. Different lowercase letters indicate differences across treatments, while different capital letters indicate differences among experimental setups. Values represent means ± SEM

To contextualize these findings, root exudation data from the 28 °C hydroponic and SCE were compared with those from field-grown maize plants reported by Santangeli et al. (2024). Field samples were collected at BBCH growth stages 14 and 19, which were selected because they bracketed the biomass values observed for the laboratory experiments under 28 °C day/21 °C night settings (Fig. 5b, c).

Exudation rates from hydroponic plants grown at 28 °C were significantly lower than those observed in the field. In contrast, exudation rates from the SCE 28 °C were not significantly different from those measured in field-grown plants at either BBCH stage (Fig. 5a).

Notably, *rth3* plants exhibited a higher exudation rate in the field compared to WT, and a similar trend was observed in the SCE system. However, in the SCE, this difference was not statistically significant (p = 0.10) (Fig. 5a). Overall, the general trends and gross exudation rates were consistent across all the soil-based growth systems.

## Discussion

### Experimental system-dependent variation in root carbon exudation in maize

This study revealed that in maize, root C exudation rate and plant growth performances are strongly influenced by the experimental system adopted to cultivate them. Although the experiments were designed to align plant developmental stages across systems, both root and shoot biomass differed significantly between hydroponic and SCE (Fig. 2b, c), suggesting variations in developmental progression. This occurred despite all plants having between two and three fully developed leaves at the time of sampling, which indicates comparable developmental stages. Nevertheless, even such subtle developmental differences may have contributed to the observed variation in C exudation rates across systems.

In addition to developmental stage, methodological factors may also have influenced exudation measurements. As shown by Otxandorena-Ieregi et al. (2024), the root to sampling volume ratio (RSVR), defined as the proportion of the root system exposed to the exudate sampling solution, had a significant impact on measured C exudation rates. Because root exudation is dominated by passive transport driven by the concentration gradient (Canarini et al. 2019; Nguyen 2003), a lower RSVR is expected to result in a greater concentration gradient, potentially leading to an overestimation of the exudation rate. In our study, plants grown hydroponically at 21 °C had a significantly lower root biomass (Fig. 2c), resulting in a significantly smaller root to sampling volume ratio compared with both SCE systems and hydroponic 28 °C (Fig. 3), which likely inflated the measured C exudation rate in this system.

Similarly, the interpretation of results from the soil-REC system introduces its own set of methodological considerations. Designed to collect exudates from soil-grown plants with minimal interference, this system maintains close contact between the root and soil compartments while preventing direct root penetration into the soil (Oburger et al. 2013). Its unique design enables the collection of exudates without damaging the root system, thereby reducing bias associated with excavation-based exudate sampling in soil systems (Oburger et al. 2013; Oburger and Jones 2018). However, the need to obtain a full root mat cover on the membrane surface, along with the limited plant growth achievable in the rhizobox, necessitates cultivating several plants simultaneously within a single unit (Oburger et al. 2013) (Fig. 1). This may introduce the potential for plant-plant interactions, as growing several individuals in a confined soil volume may influence exudation patterns due to competition (Scherling et al. 2010; Weinhold et al. 2022). Consequently, gross exudation values obtained from the soil-REC are not directly comparable to those from the SCE, though genotypic differences observed in this setup remain valuable for interpreting the contribution of root hairs to exudation dynamics (see Section "Contribution of root hairs to exudation depends on experimental design and growth environment").

### System-specific temperature responses in maize reveal methodological and physiological drivers of root exudation

Raising the growth temperature to 28 °C during the day (21 °C at night) provided further insights into how environmental context and experimental system interact to shape exudation dynamics. As expected, growth temperature had a strong influence on plant biomass, with both shoot and root biomass being significantly higher at 28 °C compared to 21 °C in both hydroponic and SCE systems (Fig. 4b, c). These observations align with previous studies showing that higher temperatures, if not extreme to compromise root growth, generally promote plant growth (González-García et al. 2023; Ohtaka et al. 2020). Interestingly at 28 °C, shoot biomass did not significantly differ between SCE and hydroponic systems (Fig. 4b), suggesting that hydroponic setups, under optimal conditions, can support aboveground growth like soil-based systems.

In contrast to the consistent biomass increase with temperature, C exudation responded differently depending on the system. In the hydroponic setup, exudation rates decreased at 28 °C compared to 21 °C, while in the soil-based system, it increased under the warmer conditions (Fig. 4a). This opposing response resulted in a highly significant temperature × experimental system interaction, explaining 64% of the total variation in C exudation (*p* < 0.001, Table 1). These findings highlight experimental conditions strongly influence physiological responses and illustrate the challenges of interpreting exudation data across different experimental setups.

The increase in C exudation rate in SCE at 28 °C aligns with previous research suggesting that warming can stimulate root exudation as plants need to increase nutrient mobilisation to support higher biomass (Wang et al. 2021). In line with this consideration, our study showed that plants grown at 28 °C in the SCE had both higher biomass and increased exudation rate. In addition, SCE at 28 °C combined relatively higher specific root length with a reduced root-to-shoot ratio, suggesting a temperature-driven shift toward faster shoot growth alongside a more resource-explorative root strategy (i.e., finer roots with higher length per unit biomass) to meet increased nutrient demand. Notably, when RSVR was comparable between the hydroponic and SCE systems (Fig. 3), the SCE 28 °C showed the highest C exudation rate (Fig. 4a). In contrast, the lower RSVR observed for hydroponic 21 °C (Fig. 3) suggests that the elevated exudation rate at 21 °C in hydroponics may represent a methodological artefact, while the increased exudation in SCE 28 °C more likely reflects a true physiological response. This highlights the importance of considering the RSVR when investigating exudation, as discrepancies in this factor can lead to misleading comparisons across experimental systems or growth conditions.

To assess the ecological relevance of these experimental results, we compared laboratory data from the 28 °C experiments with exudation rates obtained from field-grown maize sampled at BBCH stages 14 and 19 (Santangeli et al. 2024). The laboratory temperature regime (28 °C day/21 °C night) was chosen to approximate the field conditions experienced by plants at BBCH 14, based on temperature records from Jorda et al. (2022), and Vetterlein et al. (2022). Although daily mean temperatures were similar at the time of exudate sampling in the field, early field growth was characterised by cold weather, which is likely to have delayed plant development (Vetterlein et al. 2022). Therefore, including data from BBCH 19 allowed a more meaningful comparison, as the plant biomass from the hydroponic and SCE at 28 °C fell between those observed at BBCH 14 and 19 (Fig. 5b, c), providing a more accurate representation of growth dynamics across experimental conditions. Further, RSVR values for the SCE 28 °C were comparable to those observed in field samples at BBCH 14, with no significant genotypic differences (Fig. 3). While BBCH 19 plants exhibited slightly higher RSVR values, these remained within the acceptable range proposed by Otxandorena-Ieregi et al. (2024), supporting their inclusion in the study.

Comparing laboratory results with field data revealed clear outcomes. C exudation rates in hydroponic plants grown at 28 °C were significantly lower than those in the field, regardless of the genotype (Fig. 5a). In contrast, SCE 28 °C exudation rates did not differ significantly from those of field-grown plants at either BBCH stage (Fig. 5a). This indicates that soil-based laboratory systems can approximate field exudation dynamics when key factors such as temperature and plant developmental stage are comparable. In this context, soil-based setups represent a useful and valid platform for investigating exudation processes.

Despite similar shoot biomass across systems (Fig. 4b), a lower exudation rate in hydroponics can be expected, as the absence of a solid substrate has already been shown to affect plant metabolism (Atwell 1993), alter root morphology and reduce exudation in maize plants (Boeuf-Tremblay et al. 1995; Groleau-Renaud et al. 1998). In line with this, hydroponic systems in the present study generally resulted in smaller average root diameters and lower root-to-shoot ratios compared with soil-based systems (Fig. S2), consistent with the lack of mechanical impedance and homogeneous nutrient availability in solution. The ready availability of nutrients in the solution further reduces the need for belowground carbon investment (Wang et al. 2021), as reflected in the lower root biomass and root C exudation rate observed in the hydroponic setup (Fig. 4a, c). Consequently, hydroponic systems may underestimate both the rate and ecological role of root exudation. Supporting this, Otxandorena-Ieregi et al. (2024) reported approximately 40% lower exudation in hydroponically grown maize compared to soil-grown plants under identical conditions. In the present study, hydroponic plants exuded roughly 50% less C, on average across genotypes, than plants grown in the SCE (Fig. 5a). These findings are particularly relevant as they demonstrate that the differences between hydroponic and soil-based systems extend beyond differences in metabolite composition, complementing the observations made by Heuermann et al. (2023), and highlight limitations of hydroponic systems in representing natural root exudation processes.

### Contribution of root hairs to exudation depends on experimental design and growth environment

Our results showed genotypic differences in both plant growth and carbon (C) exudation, with *rth3* consistently exhibiting reduced shoot and root biomass compared to WT across soil-based systems and temperatures (Figs. 2, 4, 5). These results align with our expectations and with previous findings, where the *rth3* mutant was reported to have reduced growth compared to WT (Hochholdinger et al. 2008; Lippold et al. 2021; Lohse et al. 2023; Santangeli et al. 2024; Vetterlein et al. 2022). However, while biomass differences were generally consistent, the genotypic differences in C exudation varied depending on environmental conditions and the experimental system.

Notably, no genotypic differences were observed in the hydroponic system, regardless of temperature. The absence of mechanical impedance may influence the development or functionality of root hairs in the WT plants (pers. comm. Frank Hochholdinger). Although microscopy images confirmed that WT plants developed root hairs under hydroponic conditions (Fig. S1), their functionality in nutrient uptake under this setup remains uncertain, potentially masking differences between the two genotypes examined in this study. Additionally, nutrient availability in hydroponics is typically non-limiting, reducing the physiological need for belowground C allocation (Wang et al. 2021), which could further mitigate genotypic contrasts.

Conversely, the similar exudation rate between WT and *rth3* observed in the SCE 21 °C was unexpected. While some differences might be anticipated when comparing laboratory and field experiments due to the unpredictable combinatorial effects of several environmental stimuli (Heuermann et al. 2023; Oburger and Jones 2018; Santangeli et al. 2024), the experiment reported in Lohse et al. (2023) used the same experimental setup, plant genotypes, and sampling protocol as in the present study. The contrasting results likely stem from differences in growth conditions the plants experienced. Specifically, Lohse et al. (2023) conducted the SCE in a growth chamber equipped with sodium-vapour lamps, whereas the SCE of this study was conducted in a growth cabinet fitted with LED lights. Despite targeting the same light intensity (i.e., PAR 350 µmol m^−2^ s^−1^), the differing light spectra and the associated heat production may have differently influenced plant development and growth (Chiang et al. 2020; Yang et al. 2022), as reflected in the lower plant biomass observed in the SCE 21 °C (Fig. 2b, c) compared to the SCE of Lohse et al. (2023).

Beyond differences in root morphology and biomass, the higher exudation rate observed in *rth3* may also reflect an alternative C partitioning strategy. The absence of fully developed root hairs in the *rth3* plants may have reduced nutrient uptake, which the plant may compensate for by a greater investment in root growth or exudation (Vetterlein et al. 2022), to potentially mobilize nutrients or recruit beneficial microbes. This compensatory mechanism may be more pronounced under high nutrient demand (e.g., 28 °C), whereas at lower growth temperatures (e.g., 21 °C), the reduced metabolic activity of the plant could decrease the need for such a response. Supporting this interpretation, a significant three-way interaction among genotype, temperature, and experimental system (p = 0.02), though explaining a relatively small portion of the variance (3.6%) (Table 1), reinforces that the effects of root hairs on exudation are highly context dependent. Furthermore, Ganther et al. (2022) found no difference in the amount of freshly assimilated ^13^C in shoot or root tissues between field-grown *rth3* and WT plants, suggesting that the higher exudation rate of *rth3* reflects a redistribution of fixed carbon rather than enhanced C assimilation (Santangeli et al. 2024). Nevertheless, whether the higher exudation reflects a compensatory mechanism for reduced root surface area (Fig. S2) or the release of surplus assimilated carbon not allocated to biomass remains unclear and requires further investigation to elucidate the underlying mechanisms.

Among the tested systems, the soil-REC setup most clearly revealed genotypic differences in exudation (Fig. 2a). Since the rhizobox-based design allows exudate collection from undisturbed roots while maintaining root-soil contact and preserving root hair integrity (Oburger et al. 2013), the soil-REC system is particularly suitable for assessing the contribution of root hairs to C exudation. Additionally, the confined soil volume and higher plant density likely increased nutrient competition between plants, which may have further intensified the compensatory response in *rth3*.

Finally, although the soil-hydroponic hybrid approach used in both SCE and field experiments may lead to partial root hair loss during sampling, previous root scanning showed that root hairs were still present post root washing (Santangeli et al. 2024; Fig. S1), suggesting that their contribution to exudation was still, at least partially, preserved. These results indicate that minor physical disturbances during sampling are unlikely to override the dominant influence of environmental growth conditions.

Overall, our findings highlight that genotypic differences in root exudation, such as those arising from the absence of root hairs, only become apparent under specific conditions, i.e., adequate temperature, soil-based growth, and system designs that allow for functional root hairs development.

## Conclusion

The differences in root carbon exudation observed between the experimental systems and growth conditions investigated in this study highlight the need to carefully consider the experimental design when interpreting root exudation data. The complex interactions between plant physiology and the environmental factors to which the plants are exposed will ultimately influence the root exudation dynamics. Nevertheless, the similarity between soil-based laboratory and field results indicates that exudation patterns are consistent when environmental conditions, particularly growth substrate and temperature, are comparable. These findings offer a valuable basis for improving carbon allocation models, providing robust indicators for simulating carbon fluxes in agricultural ecosystems.

In addition, the results of this study showed a strong interaction between experimental design and environmental factors in the genotype-specific carbon exudation response, suggesting that these patterns can only be accurately identified under appropriate environmental conditions and thorough selection of appropriate experimental setups. Further metabolomic studies may help to determine whether the exudation of specific metabolites is experimental-system dependent.

Our findings also confirm the importance of using similar root to sampling volume ratios (RSVR) when aiming at comparing root exudation within or across experiments, as large differences in RSVRs were shown to bias the results.

Overall, this study provides important insights into the methodology of assessing root carbon exudation and emphasises the importance of soil-based systems for generating field-relevant data that informs models of plant-soil carbon dynamics.

## Supplementary Information

Below is the link to the electronic supplementary material.

ESM1 (PDF 640 KB)

## Data Availability

The data generated during and/or analysed during the current study are available from the corresponding author on reasonable request.

## Abbreviations

DOC: Dissolved Organic Carbon
SCE: Soil Column Experiment
REC: Root Exudate Collector
DI: Deionized water
RSVR: Root to sampling volume ratio
MQW: Milli-q water
