# Supplementary material for: Comparative assessment of root exudation in maize: Influence of experimental setup, growth conditions and root hairs
Source: Plant Soil. 2026 Feb 5;520(2):1231–47. doi: 10.1007/s11104-026-08324-x (PMC13065546; doi:10.1007/s11104-026-08324-x)
Supplement: Supplementary file 1 — (PDF 640 KB) [file 11104_2026_8324_MOESM1_ESM.pdf]

Supplementary information

Plant and Soil

Comparative assessment of root exudation in maize: Influence of experimental setup, growth conditions and root hairs

Michael Santangeli[a*], Anna Heindl[a], Lisa Stein[a], Alice Tognacchini[a], Eva Oburger[a]

[a] BOKU University, Department of Ecosystem Management, Climate and Biodiversity, Tulln an der Donau, 3430, Austria

* Corresponding author: michael.santangeli@boku.ac.at

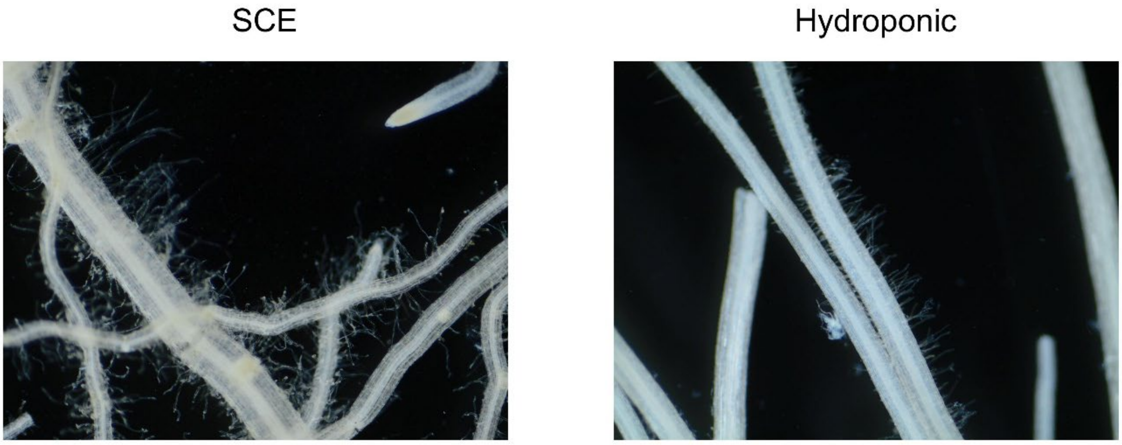

Fig. S1 Representative microscope images of maize WT roots from the hydroponic and soil column experiments (SCE) obtained after exudate sampling and WinRHIZO scans.

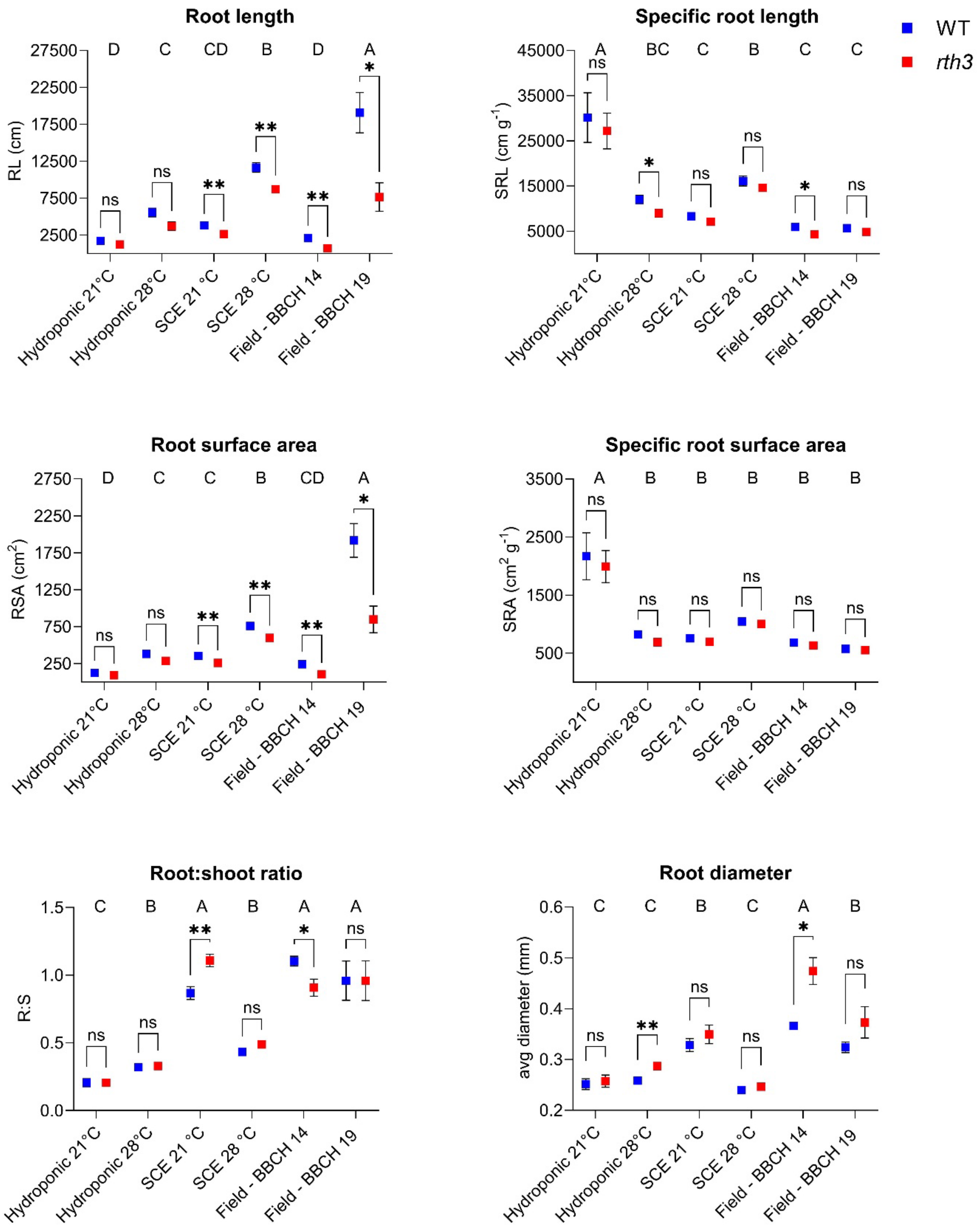

Fig. S2 Root traits of hydroponic, soil column experiment (SCE), and field data from Santangeli et al. (2024) for Wild-Type (WT) and root hairless (*rth3*) mutant maize plants. The root-to-shoot ratio (R:S) was calculated by dividing root dry biomass by shoot dry biomass for each individual plant. Root traits were computed as follows: Specific Root Length (SRL) was the total root length divided by root dry biomass (cm g^{-1}), and Specific Root Area (SRA) was the total surface area divided by root dry biomass (cm^2 g^{-1}). Differences between experimental setups were evaluated with 2-way ANOVA followed by Tukey's post hoc test. Differences between genotypes were evaluated by Welch t-test. *** $p < 0.001$, ** $p < 0.01$, *

$p < 0.05$, ns = not significant. Different capital letters indicate differences among experimental setups. Values represent means ± SEM
